# Sexual and marital trajectories and HIV infection among ever-married women in rural Malawi

**DOI:** 10.1136/sti.2008.033969

**Published:** 2009-03-13

**Authors:** C Boileau, S Clark, S Bignami-Van Assche, M Poulin, G Reniers, S C Watkins, H P Kohler, S J Heymann

**Affiliations:** 1 Cartagene, Montreal, Canada; 2 McGill University, Montreal, Canada; 3 Université de Montréal, Montreal, Canada; 4 Brown University, Providence, USA; 5 University of Colorado at Boulder, Boulder, USA; 6 University of California at Los Angeles, Los Angeles, USA; 7 University of Pennsylvania, Philadelphia, USA

## Abstract

**Objective:**

To explore how sexual and marital trajectories are associated with HIV infection among ever-married women in rural Malawi.

**Methods:**

Retrospective survey data and HIV biomarker data for 926 ever-married women interviewed in the Malawi Diffusion and Ideational Change Project were used. The associations between HIV infection and four key life course transitions considered individually (age at sexual debut, premarital sexual activity, entry into marriage and marital disruption by divorce or death) were examined. These transitions were then sequenced to construct trajectories that represent the variety of patterns in the data. The association between different trajectories and HIV prevalence was examined, controlling for potentially confounding factors such as age and region.

**Results:**

Although each life course transition taken in isolation may be associated with HIV infection, their combined effect appeared to be conditional on the sequence in which they occurred. Although early sexual debut, not marrying one’s first sexual partner and having a disrupted marriage each increased the likelihood of HIV infection, their risk was not additive. Women who both delayed sexual debut and did not marry their first partner are, once married, more likely to experience marital disruption and to be HIV-positive. Women who marry their first partner but who have sex at a young age, however, are also at considerable risk.

**Conclusions:**

These findings identify the potential of a life course perspective for understanding why some women become infected with HIV and others do not, as well as the differentials in HIV prevalence that originate from the sequence of sexual and marital transitions in one’s life. The analysis suggests, however, the need for further data collection to permit a better examination of the mechanisms that account for variations in life course trajectories and thus in lifetime probabilities of HIV infection.

With the development of life course epidemiology^1^ and sequence analysis in demographic research,^2^ increasing attention has been given to the impact of early life experiences on adult health. This approach has the potential to be useful for studying the AIDS epidemic because the temporal ordering and timing of individuals’ sexual and marital partnerships may be quite relevant for one’s lifetime risk of contracting a sexually transmitted infection.^3^

Most previous research has focused on the relationship between a single transition and HIV infection. Several studies have shown, for example, that early sexual debut is associated with an increased likelihood of HIV infection.^4^^–^6 The time between first sex and first marriage, as well as the number of premarital sexual partners, are also both believed to increase the HIV risk substantially.^3^ ^7^ The transition into marriage itself influences HIV risks: women who marry before the age of 20 are more likely to be HIV-positive than unmarried women of the same age, both because marriage typically coincides with a dramatic increase in the frequency of sexual activity and because husbands are generally older than boyfriends and thus have had more opportunity to become infected themselves.^8^ ^9^ Marriage duration may either offer protection or exacerbate the HIV risk. If both spouses enter the marriage HIV-negative and remain sexually exclusive, marriage affords considerable protection. If, however, either spouse is already infected or is subsequently unfaithful and condom use within marriage is low, frequent unprotected sex with a spouse increases the likelihood of HIV transmission.^8^^–^13 Finally, transitions out of marriage via divorce or widowhood are strongly associated with HIV status.^14^ ^15^ The causal direction of this association may be difficult to disentangle, however. For example, divorce may be provoked by suspicion of infidelity and death may be due to AIDS. Alternatively, those who are divorced or widowed may subsequently become infected in a later sexual relationship.

While previous research indicates that each of these life transitions and stages taken alone has important implications for HIV risks, life course theory emphasises that they are not independent of one another. The timing of sexual debut, for instance, marks a critical transition to adulthood that has a significant impact on future patterns of marital and non-marital relationships.^16^^–^18 Moreover, when and to whom individuals marry have a strong influence on whether or not the marriage endures. Thus, it might be fruitful to examine the relation between HIV infection and the sequence of one’s sexual and marital life transitions—that is, an individual sexual and marital trajectory. Analyses of the association between HIV infection and life course trajectories have, to our knowledge, not previously been conducted because they require detailed sociodemographic and serological panel data as well as sophisticated modelling techniques. In this paper we take a first step towards such analyses by constructing sexual and marital trajectories for 926 ever-married women in rural Malawi, and by then examining the association between these trajectories and the HIV status of the women. Although the nature of our data does not permit identification of specific causal pathways between sexual and marital trajectories and HIV infection, we are able to distinguish trajectories associated with a high HIV prevalence from those associated with a low HIV prevalence.

## METHODS

### Data and study population

The data for this study are taken from the Malawi Diffusion and Ideational Change Project (MDICP). Since 1998 the MDICP has collected panel data from ever-married women and their husbands to examine the role of social networks in changing attitudes and behaviour regarding HIV/AIDS, family size and family planning in rural Malawi (details on sampling and field work procedures, as well as the survey data, are available from the MDICP website: http://malawi.pop.upenn.edu). In the analyses that follow we use retrospectively reported data on sexual and premarital histories collected during the second wave of the MDICP in 2001, and on marital histories and HIV serology data collected in the third wave in 2004 (for details, see Bignami-Van Assche *et al*^19^).

In 2001, 1570 ever-married women completed the MDICP survey; of these, 1217 (77.5%) were re-interviewed in 2004. The majority of women who were lost to follow-up between 2001 and 2004 had moved (44.4%), were away temporarily (17.1%) or had died between the two survey waves (10.2%). Yet attrition is not likely to bias the estimates presented in this paper, as illustrated in detail elsewhere.^20^ HIV serology data were not collected in 2001, but HIV status measured from biomarker testing is available for 1024 women (84.1%) who were re-interviewed in 2004 (lack of information on HIV status is due mainly to refusal to be tested for HIV). Of the women in this latter group, 926 (90.4%) had complete survey information on sexual and marital histories and thus represent the sample for the present analysis.

### Measurement and quality of key indicators

We focus on four key events in the lives of rural Malawian women: sexual debut, premarital sexual activity, first marriage and marital dissolution via divorce (including separations) and widowhood.

*Sexual debut* was measured by the woman’s self-reported age at first sexual intercourse in the 2001 wave of the MDICP. Although it is well known that sexual activity in surveys is often underreported and/or misreported, reports about sexual activity in the MDICP seem quite reliable.^21^ ^22^

Women who reported that they did not marry their first sexual partner were inferred to have had *premarital sexual activity* (or *premarital partners*), although we have no information on the number of their premarital partners.

Retrospective self-reported information on women’s marital histories (including marital disruptions via separation, divorce or widowhood) was obtained from the 2004 wave of the MDICP. While there are different definitions of marriage across and within countries in sub-Saharan Africa, the MDICP relies on respondents’ self-reports of their status as married, regardless of whether or not there had been a public marriage ceremony. Thus, both formal and informal marriages were included.

### Methods of analysis

Descriptive statistics for the characteristics and sexual and marital histories of the selected women are presented and the bivariate associations between HIV status and these characteristics and behaviours are identified. The four key events of interest (sexual debut, premarital sexual activity, marriage and marital disruption) are then sequenced in order to construct trajectories that represent the variety of patterns in our data. To do so, we first divide the sampled women into two groups based on their age at sexual debut (before or after age 15). Second, we split each group into two subgroups according to whether the woman married her first sexual partner or not. We split each group a third time according to the outcome of the first marriage: remained married to first spouse, became divorced or separated from spouse or became widowed (women who experienced both divorce and widowhood were placed in the widowhood category). We then evaluate how HIV prevalence differs across each life course event considered individually as well as entire trajectories.

Finally, we examine the association between different trajectories and HIV prevalence controlling for potentially confounding factors such as age and region. The MDICP data were collected in three regions of Malawi (South, Centre and North) in which different religious and cultural practices are prevalent; the regions have consistently been found to differ substantially in sexual and marital patterns as well as in HIV prevalence. Specifically, we use logistic regression to model HIV status as a function of two sets of covariates. First, we examine the role of each trajectory, controlling only for age and region. Second, we add other salient characteristics of women’s sexual and marital histories: the age difference between the woman and her first spouse; whether the woman or any of her spouses had extramarital partnerships, both as reported by the woman; whether the woman had been in at least one polygamous union during her entire marriage history; and the woman’s total number of lifetime partners (both spousal and non-spousal). Covariates are introduced in the model one set at a time. Variables in the full model were selected by using a conditional stepwise backward strategy in which the statistical criteria for entry and retention of variables in each model are p⩽0.05 and p⩽0.10, respectively. Results are presented as odds ratios (OR) with 95% confidence interval (CI). All analyses were done with SPSS Version 9 (SPSS, Chicago, Illinois, USA, 2008).

## RESULTS

### Characteristics of women’s sexual and marital histories

Table 1 presents descriptive statistics for the respondents’ background characteristics and sexual and marital histories, for the characteristics and behaviours of their sexual and marital partners, and for the HIV prevalence associated with these characteristics and behaviours.

**Table 1:** Descriptive statistics and bivariate analysis for the background characteristics and sexual and marital histories of the selected women (Malawi Diffusion and Ideational Change Project, 2001 and 2004)

	Total number (%)(n = 926)	No (%) with HIV(n = 83)	p Value
Background characteristics			
Age (years)			
⩽30	241 (26.0)	22 (9.1)	0.749
31–40	339 (36.6)	33 (9.7)	
>40	346 (37.4)	28 (8.1)	
Region			
North	322 (34.8)	20 (6.2)	0.022
Central	289 (31.2)	24 (8.3)	
South	315 (34.0)	39 (12.4)	
Sexual history			
Sexual debut			
Sexual debut by age 15	371 (40.1)	41 (11.1)	0.069
Sexual debut after age 15	555 (59.9)	42 (7.6)	
First sexual partner			
Spouse	621 (67.1)	46 (7.4)	0.018
Other	305 (32.9)	37 (12.1)	
Total number of lifetime partners*			
1	458 (49.5)	23 (5.0)	<0.001
2	286 (30.9)	34 (11.9)	
⩾3	181 (19.6)	26 (14.4)	
Marital history			
Marital status in 2004			
Married	823 (88.9)	67 (8.1)	0.046
Divorced/separated	59 (6.3)	9 (15.3)	
Widowed	44 (4.8)	7 (9.0)	
Ever married	926 (100.0)		
History of divorce			
Ever divorced	328 (35.4)	43 (13.1)	0.001
Never divorced	598 (64.6)	40 (6.7)	
History of widowhood			
Ever widowed†	91 (9.8)	21 (23.1)	<0.001
Never widowed	835 (90.2)	62 (7.4)	
Characteristics and behaviours of sexual and marital partners			
Age difference with first spouse‡			
>5 years	285 (30.8)	19 (6.7)	0.202
⩽5 years	518 (55.9)	48 (9.3)	
Was part of at least one polygamous union			
Yes	458 (45.5)	50 (10.9)	0.037
No	468 (50.5)	32 (7.0)	
Had EMSP during any marriage			
Yes	79 (8.5)	9 (11.4)	0.429
No	847 (91.5)	74 (8.7)	
Knows at least one husband had EMSP§			
Yes	387 (41.8)	41 (10.7)	0.218
No	539 (58.2)	32 (8.0)	

EMSP, extramarital sexual partner(s).

*Includes both spousal and non-spousal partners.

†Includes women who were ever widowed and also ever divorced.

‡123 missing values.

§141 missing values.

Beginning with their premarital histories, 40.1% of women reported that their sexual debut occurred before the age of 15 years, with the median age at first sexual intercourse being 16 years (not shown). About one-third (32.1%) of all women reported not marrying their first sexual partner. In addition, half of all women reported having had only one lifetime sexual partner, 35.4% two partners, and 19.8% three or more partners.

With regard to the women’s marital histories, the median age at first marriage was 18 years (not shown). Most women (88.9%) were still married in 2004, 6.4% were divorced or separated and 4.8% were widowed. During their married life, 35.4% of women had been divorced at least once and 9.8% had been widowed at least once. Approximately one-third (30.8%) of all women said that their first husband had been more than 5 years older when they got married, although 13.3% did not provide age difference information. Due to sampling strategies described elsewhere,^23^ the number of women reporting having ever been in a polygynous union was high at 50%. Extramarital sexual activity was infrequently reported by women, but nearly half said they knew that at least one of their husbands had had extramarital partners while they were married.

Overall, the prevalence of HIV in 2004 for the women included in the study was 9%. Differentials in HIV prevalence by age were not statistically significant. As found in other studies,^24^ differentials by region indicate a significantly higher HIV prevalence in south Malawi (12.4%) than in north Malawi (6.2%; p<0.05). Overall, women who had an early sexual debut (that is, by age 15) had a higher HIV prevalence (11.1%) than women who had a late sexual debut (7.6%), although the difference was not highly significant (p<0.10). On the other hand, whether sexual debut occurred with a spouse rather than with another partner and the total number of lifetime partners were significantly associated with a lower HIV prevalence. Women who were still married in 2004 had a significantly lower prevalence of HIV than women who had become widows (8.1% vs. 9.0%) and, especially, than women who had got divorced (15.3%; p<0.05). A history of divorce and/or widowhood was one of the characteristics of women’s marital histories most significantly associated with a high prevalence of HIV. Finally, of the other attributes of women’s marital histories considered, only a history of polygamy was significantly associated with increased HIV prevalence.

### Women’s sexual and marital trajectories

**Figure 1:**
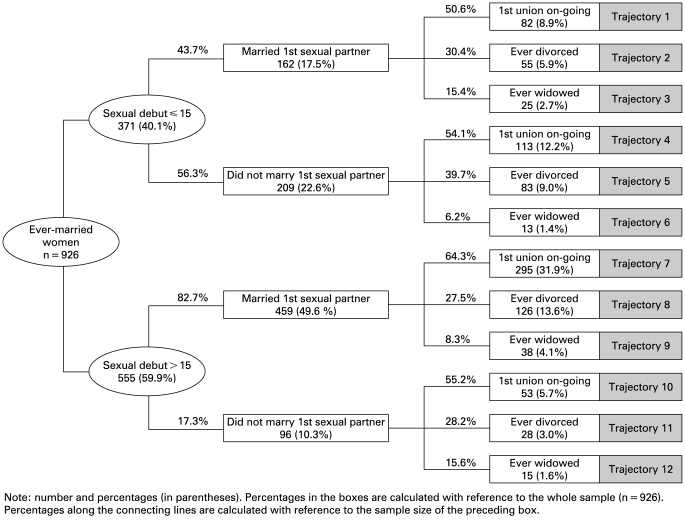
Sexual and marital trajectories of the women selected for the study, Malawi Diffusion and Ideational Change Project, 2001 and 2004. Numbers and percentages (in parentheses).

Using information on sexual debut, premarital sexual activity, marriage and marital dissolution, we identified the most common sequences or trajectories (fig 1). It is evident that an early sexual debut is associated with premarital partnerships; slightly more than half (56.3%) of the women who initiated sex at an early age reported not marrying their first sexual partner. Although the combination of early sexual debut and premarital sexual activity might be associated with greater marital dissolution, this was not the case for the women in our sample. Within the group of women who had an early sexual debut the rates of marital dissolution were remarkably similar, whether they did or did not marry their first sexual partner. Interestingly, however, the cause of the dissolution differed substantially. Over 15% of women who had sex before the age of 15 and married their first partner (ie, had an early marriage) were ever-widowed compared with about 6% of women who had sex before the age of 15 and did not marry their first sexual partner (p = 0.004). In contrast, for most women (82.7%), later sexual debut occurred with a spouse or a soon-to-be spouse and was associated with a stable first marriage: 64.3% of women in this group reported an intact first marriage (trajectory 7) compared with slightly more than 50% of women following other trajectories (trajectories 1, 4 and 10). Overall, the most common trajectory began with a late sexual debut followed by a stable marriage with the first sexual partner (31.9% of all women considered, trajectory 7).

### HIV outcomes of sexual and marital trajectories

**Figure 2:**
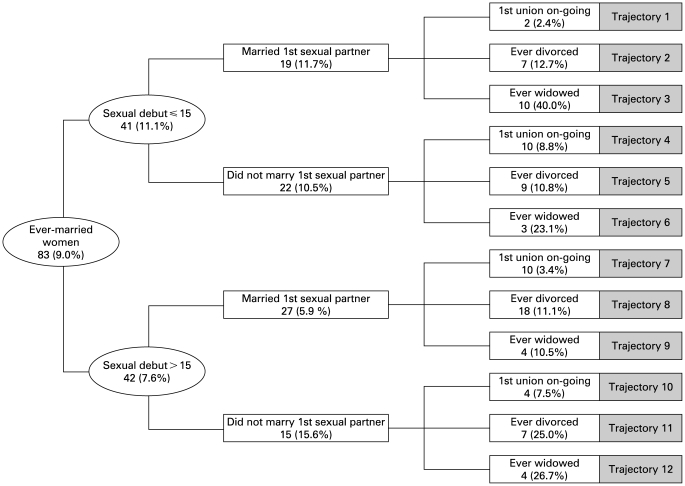
Number of HIV-positive cases and HIV prevalence (in parentheses) by sexual and marital trajectories of the women selected for the study, MDICP 2001 and 2004.

Figure 2 shows the variations in HIV prevalence according to the sexual and marital trajectories described above. Across all trajectories the prevalence of HIV was lowest among women who married their first sexual partner and remained married to that same spouse. As noted above when discussing the results in table 1, women who had an early sexual debut were slightly more likely to be infected than those who delayed it (11.1% and 7.6%, respectively; p<0.10), although the difference was only marginally significant. Among women with a sexual debut after the age of 15, those who did not marry their first sexual partner were much more likely to be HIV-positive than those who married their first sexual partner (p<0.001). Among women with a sexual debut at ⩽15 years, however, women who did not marry their first sexual partner were no more likely to be HIV-positive than women who did. This finding can be partially explained by the relatively high proportion of these young married women who are ever-widowed and that 40% of these widows are HIV-positive. Not surprisingly, widows always exhibit an increased HIV prevalence (trajectories 3, 6, 9 and 12). Ever-divorced women also had a higher HIV prevalence than women who remained in their first marriages.

The trajectories presented in figs 1 and 2 do not take into account potentially confounding factors, most notably women’s age and their place of residence. In table 2 we show the results of the logistic regression analysis of individual HIV status as a function of trajectory membership, background characteristics and sexual and marital partnership characteristics for the ever-married women in our sample. Because of endogeneity between marital dissolution and HIV status, we use an “abridged” version of the full trajectories presented in figs 1 and 2. This abridged version examines only the timing of sexual debut and whether or not the respondent marries her first sexual partner.

**Table 2:** Multivariate logistic regression analysis of HIV infection by trajectory membership, background characteristics and sexual and marital partnership characteristics, Malawi Diffusion and Ideational Change Project, 2001 and 2004

	Model 1	Model 2
	OR (95% CI)	OR (95% CI)
Background characteristics		
Age (years)	0.99 (0.96 to 1.01)	0.98 (0.96 to 1.01)
Region		
North (reference)	1.00	1.00
Central	1.24 (0.66 to 2.35)	1.07 (0.56 to 2.06)
South	1.87** (1.02 to 3.45)	1.72** (0.91 to 3.25)
Trajectory membership		
Sexual debut ⩽15, married 1st sexual partner	1.85 (0.98 to 3.49)	1.87 (0.98 to 3.58)
Sexual debut ⩽15, did not marry 1st sexual partner	1.43 (0.76 to 2.70)	1.22 (0.63 to 2.36)
Sexual debut >15, married 1st sexual partner (reference)	1.00	1.00
Sexual debut >15, did not marry 1st sexual partner	2.59** (1.30 to 5.16)	2.52** (1.25 to 5.10)
Characteristics and behaviours of sexual and marital partners		
Total number of lifetime partners		
1 (reference)		1.00
2		2.24** (1.25 to 4.03)
⩾3		2.56** (1.34 to 4.90)
Was part of least one polygamous union		1.54 (0.93 to 2.54)
Had EMSP during any marriage		0.94 (0.44 to 2.02)
Knows at least one husband had EMSP		1.00 (0.99 to 1.00)

CI, confidence interval; EMSP, extramarital sexual partner(s); OR odds ratio.

Differences in the likelihood scores between models 1 and 2 with 3 degrees of freedom were statistically significant (difference in 2-log likelihood = 24.37; df = 5; p<0.001). Model 2 therefore provides a better fit.

**p<0.05.

Model 1 evaluates the impact of trajectory membership on HIV status controlling for age and region of residence. The combination of delaying sexual debut and marrying one’s first sexual partner was associated with the lowest odds of being infected with HIV. Women who delayed sexual debut and did not marry their first sexual partner had the highest odds of being HIV-positive (OR 2.59; p<0.05). These findings provide an important insight: it appears that neither delaying sex nor avoiding having a premarital partner alone offers a clear protective advantage, but rather their effects are conditional on each other.

Controlling for sexual and marital partnership characteristics (model 2) only slightly reduces the magnitude of the effect noted above, but not its significance (OR 2.52; p<0.05). We also found, as others have,^25^ ^26^ that the number of lifetime partners (both spousal and non-spousal) was an important covariate of HIV risk net of all other factors. Indeed, the number of lifetime partners had a stronger impact on HIV risk than following any of the trajectories in the model, although these experiences were clearly interrelated.

## DISCUSSION

We have explored the links between sexual and marital trajectories and HIV status among ever-married women living in rural Malawi. Our first set of findings (presented in fig 1) shows that women’s sexual and marital transitions are linked over their life course. Of all the women, those who delay sex and marry their first sexual partner are the most likely to have a stable first marriage. In contrast, women who become sexually active before the age of 15 and marry their first partner have exceptionally high levels of widowhood. Even though our trajectories do not control for women’s age or marriage duration, sexual and marital transitions are clearly trajectory-dependent, although they are usually treated as independent factors in HIV research.

**Take-home messages**

- A life course approach was used to evaluate the relationship between HIV infection and different sexual and marital trajectories for women in rural Malawi.
- Important transitions in women’s sexual and marital lives are not independent from one another and thus should not be considered in isolation.
- Women who delayed sexual debut and did not marry their first partner are, once married, more likely to experience marital disruption and to be HIV-positive.
- Women who marry their first partner, but who have sex at a young age, are more likely to be widowed and are also at considerable risk.

Our second set of findings indicates that different sexual and marital trajectories are related to current HIV status. Consistent with other research,^3^^–^6 we found a weak bivariate association between early sexual debut and a higher likelihood of HIV infection, and a strong bivariate association between premarital partners (other than one’s spouse) and being HIV-positive. Finally, in accordance with other studies showing an association between marital disruption and HIV,^14^ ^15^ we found that, regardless of their premarital sexual histories, women who are in their first marriage were the least likely to be HIV-infected. This suggests that future research should focus on identifying the direction of the causal relationship between a disrupted marriage and HIV infection.^14^

Our main conclusion is methodological: important transitions in the lives of women are not independent and thus should not be considered in isolation. Although early sexual debut, marrying one’s first sexual partner and having a disrupted marriage each increase the likelihood of HIV infection, as reported by others,^3^–^6^ ^14^ ^15^ the risk is not additive. The strongest evidence for this conclusion comes from our multivariate regression analyses (presented in table 2). We found a clear interaction between delaying sexual debut and premarital partnerships. Nearly half of the women in our sample delayed sex and married their first partner, which is the trajectory associated with the lowest prevalence of HIV. Simply delaying sexual debut past the age of 15 alone, however, is not sufficient protection: women who delay sexual activity but do not marry their first partner are the most likely to become infected. This result reflects both the higher rates of divorce and widowhood as well as the higher rates of HIV among those divorced and widowed in this group (see trajectories 11 and 12). Similarly, marrying one’s first sexual partner does not always reduce the HIV risk. Conditional on early sexual activity, women who married their first sexual partner were more likely to be HIV-positive than those who did not. Since many of the women in this category are young brides with older husbands, it is not surprising that over 15% become widows. What is more surprising is the rate of HIV infection among these early marrying widows. An astonishing 40% were HIV-positive, representing the trajectory associated with the highest levels of HIV (trajectory 3). These findings highlight the complex and dynamic relationships between life transitions and HIV risks, which are often inadequately captured by focusing on individual transitions rather than on their sequence.

One of the main limitations of our study is that it relies on self-reported retrospective data that may be subject to a variety of biases including problems in reporting dates with accuracy, the selective omission of unsuccessful or short unions, and social desirability bias when reporting on the occurrence and timing of sexual partnerships. Our results may also be prone to selection bias because women who were interviewed in 2001 but could not be re-interviewed in 2004 were slightly more likely to have already been divorced or widowed. Since HIV testing of MDICP respondents began in 2004, we cannot determine whether HIV prevalence among women lost to follow-up in 2004 was different from HIV prevalence among the women who were successfully re-interviewed. As a consequence, we cannot fully determine the extent of this bias. We verified, however, that there was no significant difference in sexual and marital histories between women who accepted to be tested for HIV in 2004 and those who refused. Moreover, since our results rely on a sample of ever-married women, we cannot make direct comparisons with women who never married. Finally, limitations of the data used for the analysis include the fact that HIV status was based on prevalent rather than incident infection, so we cannot make causal inferences about HIV infection and sexual and marital trajectories, particularly with respect to marital dissolution. It is unlikely, however, that HIV is an important determinant of the timing of sexual debut or of having premarital partners since a very small percentage of individuals in rural Malawi are aware of their HIV status.

Despite these limitations, our findings indicate the potential of a life course perspective for providing insights into the sources of variation in HIV infection. Sexual debut, marriage and marital disruption all occur over time, as does HIV infection; what we have shown is that the sequence matters. The full potential that a life course perspective brings to illuminate the mechanisms that determine distinct sexual marital trajectories and differences in the lifetime probabilities of HIV infection, in Malawi as elsewhere in sub-Saharan Africa, cannot be exploited without collecting new data that permit establishing the timing of HIV infection in the life course of individuals.
